# Genetic correlation and causal relationship between sleep and myopia: a mendelian randomization study

**DOI:** 10.3389/fgene.2024.1378802

**Published:** 2024-07-09

**Authors:** Guandong Zhu, Ruikang Tian, Dengke Zhou, Xuejiao Qin

**Affiliations:** ^1^ Department of Ophthalmology, The Second Hospital of Shandong University, Jinan, China; ^2^ Eye Centre of Shandong University, Jinan, China; ^3^ School of Ophthalmology and Optometry and Eye Hospital, Wenzhou Medical University, Wenzhou, China; ^4^ State Key Laboratory of Optometry, Ophthalmology, and Vision Science, Wenzhou, China; ^5^ The No. 1 Hospital of Xi’an, Xi’an, China

**Keywords:** myopia, sleep duration, chronotype, insomnia, mendelian randomization, causal analysis using summary effect

## Abstract

**Purpose:**

To investigate the genetic correlation and causal links between sleep traits (including sleep duration, chronotype, and insomnia) and myopia.

**Methods:**

Summary data on three sleep traits (sleep duration, chronotype and insomnia) and myopia from FinnGen (n = 214,211) and UK Biobank (n = 460,536) were analyzed using linkage disequilibrium score regression (LD Score), univariable and multivariable mendelian randomization (MR) experiments and Causal Analysis Using Summary Effect (CAUSE) estimation.

**Results:**

LD Score regression detected candidate genetic correlation between sleep traits and myopia, such as sleep duration, chronotype (Genetic Correlation Z-score >10.00, h2*_*observed_*p* < 0.005, Lambda GC > 1.05, *p* > 0.05). Univariable MR analyses indicated that increased sleep duration has a promotional effect on the occurrence of myopia (*p* = 0.046 < 0.05, P_FDR = 0.138 < 0.2, OR = 2.872, 95% CI: 1.018–8.101). However, after accounting for potential confounding factors, multivariable MR and CAUSE analysis did not provide evidence for a causal effect of the three sleep traits on myopia.

**Conclusion:**

There may be a potential genetic correlation between sleep duration, chronotype and myopia. However, neither of sleep duration, chronotype or insomnia had causal effect on myopia.

## 1 Introduction

Myopia, commonly known as nearsightedness, occurs when the eyeball is elongated, causing distant objects to be focused in front of the retina, resulting in blurry vision (Baird et al., 2020). This condition is a leading cause of vision impairment andblindness, significantly impacting public health and the economy, especially in Eastand Southeast Asia (Holden et al., 2016). Exploring the risk factors for myopia using various approaches will enhance our comprehension of myopia's underlying mechanisms, thereby aiding in the reduction of its current high incidence (Morgan et al., 2021).

Recent studies have unveiled that myopia is escalating globally due to a complex interplay of genetic and environmental factors. There is strong evidence that various sleep characteristics, such as sleep latency (Kocevska et al., 2021), sleep duration (Dashti et al., 2019), and insomnia (Lane et al., 2019), are shaped by genetic factors. The relationship between sleep and myopia has been controversial in observational massive crowd research for nearly 3 decades (Table 1) (Zhou et al., 2015; Ayaki et al., 2016; Jee et al., 2016; Sensaki et al., 2018; Burfield et al., 2019; Ostrin et al., 2020; Wei et al., 2020; Rayapoullé et al., 2021; Li et al., 2022; Stafford-Bell et al., 2022; Lin et al., 2023). Previous studies have reported varying findings, with some indicating a significant association between sleep traits such as duration and myopia. For instance, Jee revealed that the refractive error increased by 0.1D per 1 h increase in sleep after adjusting for confounders. Another study found that sleep time less than 7 h per day was the risk factor. On the other hand, some studies, such as Wei’s, have found no significant correlation between sleep duration and bedtime with myopia. These inconsistencies may stem from methodological differences, population heterogeneity.

**TABLE 1:** Summary of the findings between sleep and myopia.

Research Titles	Year	Author	Ethnicity	Sleep evalution		Myopia evalution		Findings
				Assessment method	Respondent	Definition of myopia	Cycloplegic refraction	
Decreased sleep quality in high myopia children	2016	Ayaki et al	Japanese	Pittsburgh Sleep Quality Index (PSQI), Hospital Anxiety and Depression Scale (HADS),representative questions from morningness/eveningness questionnaire	Participants	SE ≤ −0.50D in the worse eye	No	The results revealed that high myopia errors were significantly correlated with poor PSQI scores (*p* < 0.05), short sleep duration (*p* < 0.01), and late bed time (*p* < 0.01)
Inverse relationship between sleep duration and myopia	2016	Jee et al	Korean	Health Interview	Participants	SE ≤ −0.50D in the right eye	No	The refractive error increased by 0.10 D per 1 h increase in sleep after adjusting for potential confounders. However, high myopia was not associated with sleep duration
Sleep in Myopic and Non-Myopic Children	2020	Ostrin et al	**	Wearable device	N/A	Mean SE of both eyes ≤ −0.50D and one eye ≤ −0.75D	No	Findings showed that sleep latency is significantly shorter for myopic children compared with non-myopic children. Additionally, sleep duration and bedtime showed significant interactions with refractive error by season and day of the week
Longitudinal association between sleep features and refractive errors in preschoolers from the EDEN birth-cohort	2021	Rayapoullé et al	French	Questionnaire	Parents	Subjective inquiry	**	This study found a U-shaped association between sleep duration at age 2 and eyeglass prescription for refractive errors at age 5, and a linear positive association between midsleep at age 2 and eyeglass prescription at age 5
The association between sleep duration and risk of myopia in Chinese school-aged children: a cross-sectional study	2023	Lin et al	Chinese	Questionnaire	Participants	UDVA (Uncorrected distance visual acuity) ≤5.0 and SE ≤ −0.50D	No	Univariate logistic regression and multivariate analyses revealed that sleep duration was inversely associated with the risk of myopia in Chinese school-aged children
Disordered sleep and myopia risk among Chinese children	2015	Zhou et al	Chinese	Children’s Sleep Habits Questionnaire (CSHQ)	Parents	SE ≤ −0.50D in both eyes	Yes	When total sleep duration (night-time + midday sleep) was considered, the association between sleep duration and myopia risk was not significant.Odds of myopia ≤ -0.50D increased with worse CSHQ score (OR = 1.01, 95%CI = 1.01-1.02, *p* = 0.014)
Sleep Duration in Infants Was Not Associated With Myopia at 3 Years	2018	Sensaki et al	Chinese, Malay, Indian	Brief Infant Sleep Questionnaire (BISQ)	Parents	SE ≤ −0.50D in the right eye	Yes	Sleep duration and quality at 12 months of age were not associated with refractive error at 3 years
Ocular and Systemic Diurnal Rhythms in Emmetropic and Myopic Adults	2019	Burfield et al	**	Actiwatch Spectrum	N/A	SE ≤ −0.75D	No	Previous light exposure was not associated with the amplitude of diurnal variation in axial length, choroidal thickness, MOPP, body temperature, melatonin concentration, or cortisol concentration in this group of young adults
Sleep Duration, Bedtime, and Myopia Progression in a 4-Year Follow-up of Chinese Children: The Anyang Childhood Eye Study	2020	Wei et al	Chinese	Questionnaire	Parents	SE < −0.5D	Yes	There was no significant association between sleep duration and bedtime with myopia progression and axial elongation among children
Associations of 12-year sleep behaviour trajectories from childhood to adolescence with myopia and ocular biometry during young adulthood	2022	Stafford-Bell et al	Caucasian, EastAsian, South Asian, Other/Mixed	Child Behaviour Checklist questionnaire (CBCL)	Parents	SE ≤ −0.50D in either eye	Yes	There was no discernible correlation between sleep problem behaviour and alterations in refractive error, axial length, or corneal radius

Note: ** indicates no data available; N/A indicates Not Applicable; SE, spherical equivalent.

To overcome these limitations, a new model named mendelian Randomization (MR) (Burgess et al., 2015b; Burgess et al., 2017a), a method which resembles the RCT design, has recently emerged that provides some inspiration. Mendelian Randomization infers genetic causality between two phenotypes by using genetic variants that are fixed at conception and remain unchanged throughout an individual’s life. This stability means these variants are not influenced by external confounding factors such as lifestyle or environment, making them reliable proxies for the risk factors being studied and has been successfully used to study the relationship between different risk factors and myopia (Mountjoy et al., 2018; Plotnikov et al., 2020; Chong et al., 2023; Li et al., 2023). In this study, three specific sleep traits, including sleep duration, chronotype and insomnia, that may represent sleep quality and sleep problems were chosen (Madrid-Valero et al., 2020). Understanding the genetic and causal relationships between sleep and myopia is crucial in developing effective prevention and intervention strategies. The results of our study provide preliminary insights into the genetic and causal relationships between sleep and myopia, offering a novel perspective that addresses the limitations of traditional observational methods.

## 2 Materials and methods

### 2.1 Data sources

Summary statistics for the three indicators of sleep—sleep duration, chronotype, and insomnia—were obtained from the European Molecular Biology Laboratory and European Bioinformatics Institute (EMBL-EBI) GWAS data-base downloaded from GWAS Catalog (https://www.ebi.ac.uk/gwas/, the full IDs were described in Supplementary Table S1). The present study leveraged the genetic cohorts from the most extensive array of single nucleotide polymorphisms (SNPs) conducted for sleep duration and chronotype (Jones et al., 2016), and the hitherto largest GWAS conducted for insomnia (Sakaue et al., 2021). The FinnGen and the UK Biobank (https://hail.is/) provided summary statistics for GWAS on myopia (Canela-Xandri et al., 2018; Kurki et al., 2023). As this study was based on published data, no ethical approval or informed consent was required.

#### 2.1.1 GWAS summary statistics of sleep traits

A large European population-based study including 127,573 persons provided the genetic connection data regarding sleep duration (Jones et al., 2016). About 16,761,225 SNPs were analyzed in total. Extreme responses that required over 18 h of rest or fewer than 3 h of sleep in this cohort were discarded. Additionally, chronotype genetic association estimates were obtained from the same GWAS among 127,898 people of European ancestry, include 16,760,980 SNPs analyzed (Jones et al., 2016). And the genetic association of insomnia was obtained by GWAS analysis of a total of 486,627 individuals and 25,845,016 SNPs (Sakaue et al., 2021).

#### 2.1.2 GWAS summary statistics of myopia

The myopic GWAS of the FinnGen consisting of 1,640 cases and 212,571 controls, and approximately 16,380,455 SNPs were analyzed. Summary statistics of the myopic validation set, which refers to the dataset used to validate our findings, comprising summary statistics for myopia from UKB, including a large sample size and extensive genetic data to verify the robustness of our results, were collected from the hitherto largest genome-wide association studies conducted for myopia from UKB. The myopic GWAS of UK Biobank consisting of 37,362 cases and 423,174 controls, and approximately 9,851,867 SNPs were analyzed.

### 2.2 Genetic correlation estimation

**FIGURE 1:**
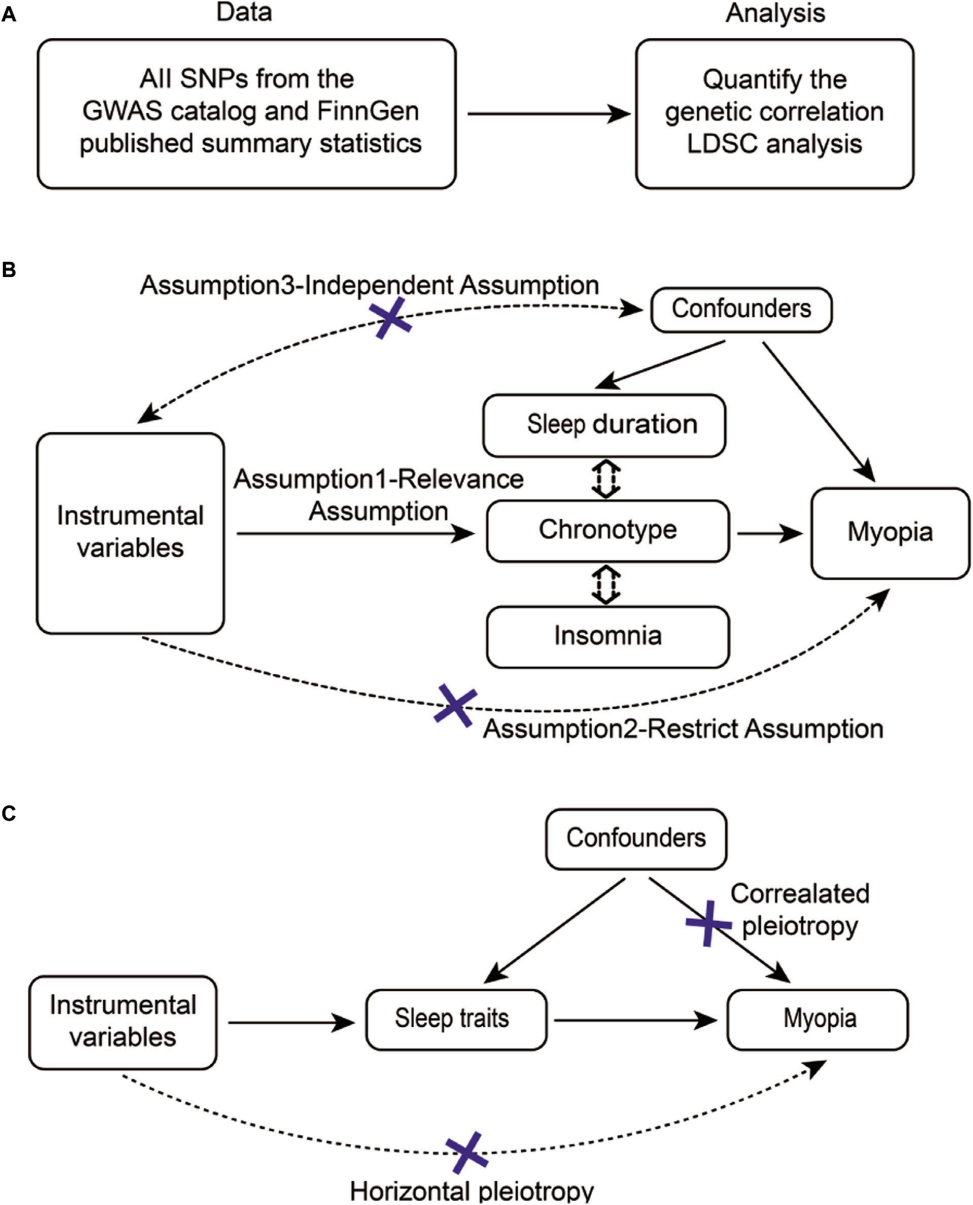
Flow diagram of the study design overview. **(A)** The flowchart of LDSC outlined eligible participants for this study. **(B)** The flowchart of MR and **(C)** CAUSE summarized sources of different confounders for each analysis. Note: SNPs, Single Nucleotide Polymorphisms; GWAS, Genome-Wide Association Study; FinnGen, Finnish Genetics of Prevalent Diseases; LDSC, Linkage Disequilibrium Score Regression.

We employed the LD Score regression (v1.0.1) to estimate the genetic correlation between sleep traits and myopia (Figure 1A) (Bulik-Sullivan B. K. et al., 2015). This method capitalizes on the fact that the GWAS effect size estimation for a given SNP incorporates the effects of all SNPs in linkage disequilibrium (LD) with that SNP, thereby providing an estimate of the genetic correlation (Bulik-Sullivan B. et al., 2015). Initially, we synchronized the effect and non-effect alleles of the GWAS datasets using the munge_sumstats.py script. This script facilitates the alignment of summary statistics from different GWAS, ensuring consistent comparison and integration of data.

Then, using the pre-generated LD scores file from the 1000 Genomes European data (Auton et al., 2015), the genetic association between sleep traits and myopia was determined using LD Score regression (https://github.com/bulik/LD Score regression). The LD Score regression analysis detected the LD Score regression intercept, observed scale heritability (Zheng et al., 2017) and genetic correlation (rug, h2_observed, Genetic Correlation Z-score and Lambda GC) between sleep traits and myopia. If the value of the Lambda GC is significantly greater than 1.05, there may be a genetic structure bias.

### 2.3 Instrumental variable selection

To ensure effective IVs, the three basic model assumptions of univariable and multivariable MR analysis should be met. First, genetic instruments for myopia and sleep traits were obtained from the above GWASs and selected independent genome-wide significant variants (significance set at *p* < 5 × 10^−8^) for each trait of interest. If the number of SNPs obtained was less than 3, *p* < 5 × 10^−6^ was used as a screening criterion. After removing the SNPs which had palindrome sequences, these SNPs were clumped based on linkage disequilibrium (*r* ^2^ < 0.001) in the given genome region, using the superpopulations “EUR” as reference panels. In addition, it was assured no interacting second phenotypes were found for the SNPs involved in the exposures in this paper. The Steiger Test was introduced to test whether the correlation between SNPs and myopia is greater than that of sleep traits, thus ruling out reverse causality (Figure 1B, Assumption2) (Hemani et al., 2017). The false discovery rates (FDR) correction is used to control the number of false positive events in multiple comparisons by correcting the *P*. The corrected *P* is called *P _FDR* and the *P _*FD*R* < 0.2 is considered statistically significant. For univariable MR analyses, using the F statistics (F = beta2/se2) for each remaining SNP, a general F statistic for all SNPs was determined (Sanderson and Windmeijer, 2016), and SNPs with lower statistical power (F statistics <10) were eliminated (Sanderson et al., 2021). The details are provided in Supplementary Tables S1–3. To satisfy the requirements of the independence assumption, we excluded some instrumental variables that are related to confounding factors. The primary method involved searching for the associations between instrumental variables and phenotypes using the PhenoScanner website (http://www.phenoscanner.medschl.cam.ac.uk/), and subsequently removing instrumental variables that were linked to confounding factors. (Supplementary Table S9).

### 2.4 Mendelian randomization analysis

All statistical analyses and data visualization were performed by R version 4.1.2 (https://www.r-project.org/). For each sleep trait, we performed Mendelian randomization analyses using the R packages “Two Sample MR” (Hemani et al., 2018)” and “Mendelian Randomization (Yavorska and Burgess, 2017)” (Supplementary Table S3). Inverse-variance weighted (IVW) method (Burgess et al., 2015a) was the primary approach used in this research to estimate the correlations between sleep traits and myopia. To ensure robustness, MR-Egger (Burgess and Thompson, 2017) and weighted median (Verbanck et al., 2018) were used as supplementary analysis methods, which help to account for potential pleiotropy and provide more reliable causal estimates. If the assumption that all included SNPs can be used as effective IVs is met, the IVW method provides an accurate estimate (Yuan and Larsson, 2020). MR-Egger regression can detect and adjust the pleiotropy. Weighted median gives an accurate estimate based on the assumption that at least 50% of IVs are valid (Bowden et al., 2016).

Then the multivariable MR (Supplementary Table S5) were conducted. In combination with previous observational studies, the multivariable MR analysis included gender, parental myopia, time outdoors, near work, education as confounding factors. Both the IVW and MR-Egger framework have been extended to estimate causal effects in multivariable MR analysis (Burgess and Thompson, 2015; Rees et al., 2017), which was conducted using both the MVMR (version 0.2.0) and Mendelian Randomization (version 0.5.0) packages in R. The mediation effect was calculated using the formula: (total effect—direct effect)/total effect and the standard error of the mediation estimate was calculated using the propagation of error method (Burgess et al., 2017b). Following the application of the Lasso method for feature selection, the coefficients derived from the Lasso results can be employed as weights within the IVW approach, thereby yielding the MR-Lasso value (Grant and Burgess, 2021).

### 2.5 Sensitivity analysis

Mendelian Randomization Pleiotropy Residual Sum and Outliers (MR-PRESSO) method was utilized to automatically identify and eliminate outliers in IVW linear regressions, thereby yielding corrected MR estimates (The “MRPRESSO” R package was used to run the MR-PRESSO). The MR-Egger intercept method was employed to test for horizontal pleiotropy of IVs, with the intercept serving as an estimate of the mean horizontal pleiotropy effect of all SNPs in the MR-Egger test., If the *p*-value is less than 0.05, there is a possibility of bias in the IVW estimate (Lawlor et al., 2008). The assessment of heterogeneity between individual SNP estimates through Cochran’s Q test provides evidence. In the event of *p* > 0.05, indicating the absence of heterogeneity (Bowden et al., 2015). Leave-one-SNP-out analysis is used to identify SNPs with potential impacts and evaluate the reliability of the results. The details are provided in Supplementary Tables S2-S4.

### 2.6 Causal analysis using summary effect estimates (CAUSE)

According to work (https://github.com/jean997/cause) of Jean Morrison et al (Morrison et al., 2020), The corresponding results for CAUSE were obtained after formatting the data for use with CAUSE (Figure 1C), calculating nuisance parameters and LD pruning (Supplementary Table S6; Supplementary Figure S1). If the result of expected log pointwise posterior density (ELPD) is negative, the causal model is more applicable between sleep traits and myopia. And the *p* < 0.05 in the casual result proves a positive result under the CAUSE method causal model. It shows the causal relationship between sleep characteristics and myopia can be proved even in the presence of horizontal pleiotropy.

## 3 Results

### 3.1 LD score regression analysis between sleep traits and myopia from FinnGen

**FIGURE 2:**
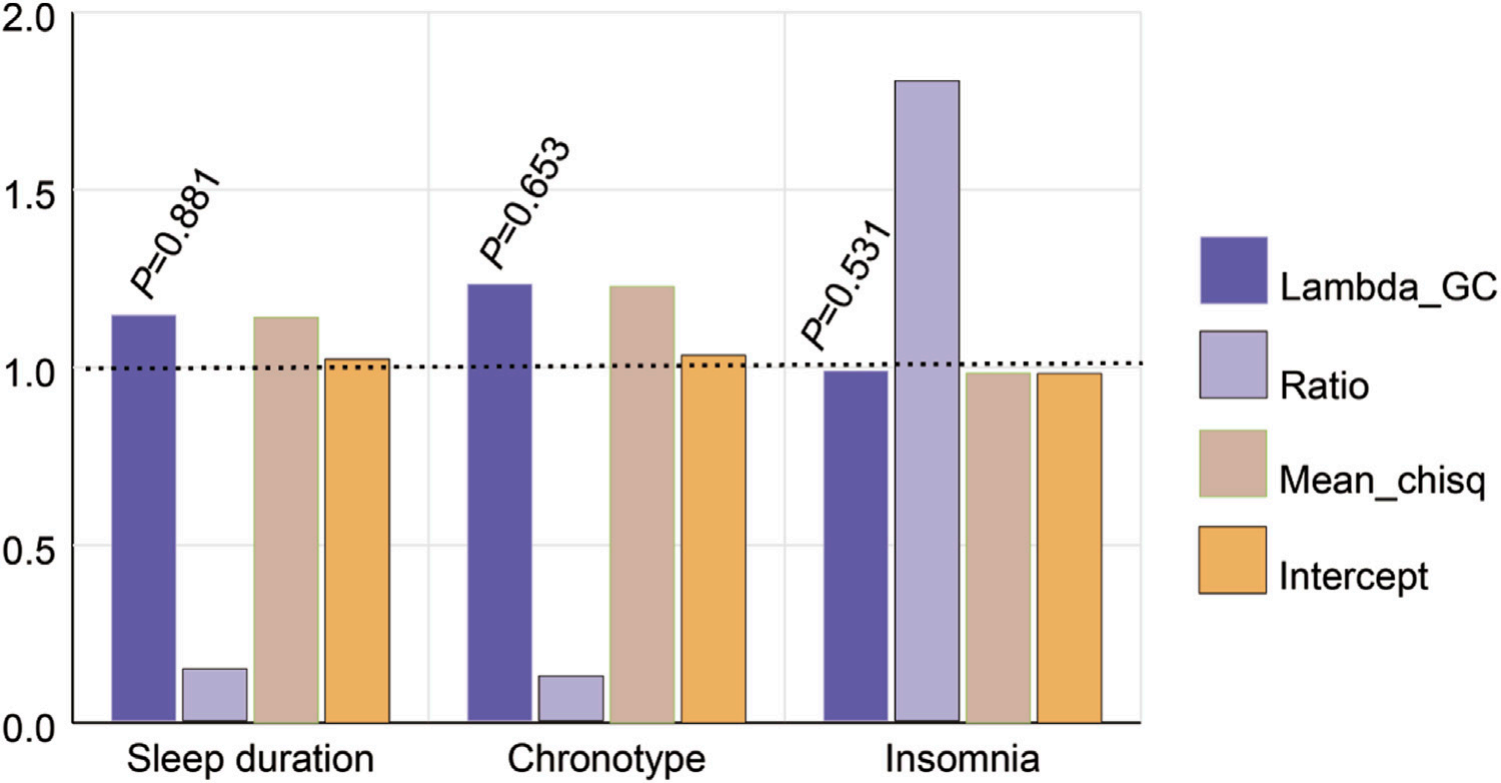
Estimated genetic correlation of sleep traits and myopia with the LDSC method. Lambda GC was used to assess genetic structural bias in GWAS, but none of them were statistically significant (*p* > 0.05). Higher ratio values indicated greater contribution of genetic factors in complex traits. Larger Mean chisq values indicated stronger associations between SNPs and target traits. The value of intercept indicated the presence of basal risk for the complex trait.

In the LD Score calculation of this study, only the heritability was statistically significant, which implies the genetic variation in sleep-related genes plays an important role in the degree or severity of myopia among individuals (Figure 2). Some extremely significant heritability (h2_observed_*p* < 0.005, Genetic Correlation Z-score >10.00) were confirmed, including sleep duration (h2_observed_*p* = 7.96 × 10^−26^, Genetic Correlation Z-score = 10.51) and chronotype (h2_observed_*p* = 1.06 × 10^−52^, Genetic Correlation Z-score = 15.28).

Gene pleiotropy and genetic correlation may not reach the threshold of statistical significance in this case. LD Score regression detected candidate genetic correlation between two sleep traits (sleep duration (Lambda GC = 1.141, *p* = 0.881) and chronotype (Lambda GC = 1.227, *p* = 0.653)) and myopia. The Lambda GC can provide important information about gene function and interactions between genes, but may not have met the requirements for statistical significance in the studies presented here (Supplementary Table S7). This does not mean that these metrics are not important, but more sample data or more precise methods may be needed to test their statistical significance.

### 3.2 Exploring the causal links between sleep traits and myopia through MR

**FIGURE 3:**
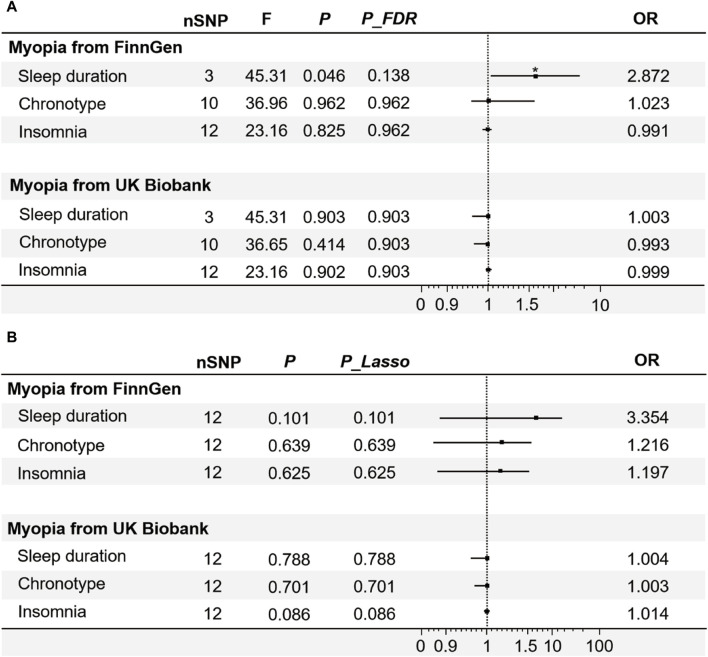
The IVW results of univariable and multivariable MR. **(A)** The results of the associations between sleep traits and myopia analysed by univariate MR, used FDR correction to control the false positive events. **(B)** The results of the associations between sleep traits and myopia analysed by multivariable MR, following the application of the Lasso method for feature selection. Note: F-statistic, used to compare the overall significance of instrumental variables; FDR, false discovery rates; OR, odds ratio. *p < 0.05.

From the univariable MR analyses (Figure 3A), only increased sleep duration has a promotional effect on occurrence of myopia from FinnGen (*p* = 0.046 < 0.05, *P_FDR* = 0.138 < 0.2, OR = 2.872, 95% CI: 1.018–8.101), which indicated that the longer an individual sleeping, the greater his/her risk of myopia (Supplementary Table S3). Based on the IVW analysis, there was no evidence to suggest a causal association between genetically predicted chronotype (*p* = 0.961, OR = 1.023, 95%CI: 0.404–2.590), insomnia (*p* = 0.825, OR = 0.991, 95%CI: 0.917–1.071) and myopia (Figure 3A). The results of other sensitivity analysis also supported the findings of the IVW analysis (Supplementary Table S3).

As a study exploring risk factors of myopia, we established a validation set to verify the reliability and scientific validity of experimental results. The data of myopia from UK Biobank was employed as a validation set for further analysis. Among the three sleep traits, no significant causal effects of the genetic instruments on myopia were observed (Figure 3A). The sensitivity analyses using median-weighted IVs was similar for all exposures (ORs were comparable to those estimated by the IVW method). Results of MR Egger were also qualitatively similar to results estimated by the IVW method (Supplementary Table S3). Sensitivity analysis showed no heterogeneity in any of the above analyses (Supplementary Table S4).

Each trait intricately linked to the others (Kocevska et al., 2021). To control for other potentially confounding variables and thus more accurately assess the effects of different sleep characteristics on myopia, an analysis of multivariable MR of the relevant GWAS data was performed. In the multivariable MR analysis about sleep traits data derived from FinnGen and UK Biobank, there was no evidence for a causal effect of sleep traits on risk of myopia using IVW (Figure 3B), MR-Egger, weighted median and MR-Lasso (Supplementary Table S5). After adjustment of other sleeping phenotypes, sleep duration had no direct effect on myopia (*p* = 0.101, Plasa = 0.101). We can tentatively infer that the occurrence of individual positive results may be due to the randomness of the allocation rather than the actual experimental effect.

### 3.3 Exploring the causal links between sleep traits and myopia through CAUSE

To improve the reliability and accuracy of the results, the CAUSE analyzing large-scale sample data was employed. The effect of sleep duration on myopia did not survive multiple comparisons correction with CAUSE (ΔELPD_*p* = 0.349, OR = 0.653, 95%CI: 0.388–1.126, Supplementary Table S6; Supplementary Figure S41A). Similarly, the CAUSE analysis supported there was no causal relationship of all sleep traits on myopia data derived from FinnGen and UK Biobank (Supplementary Table S6, in Supplementary Figures S41B–F).

The CAUSE method has two advantages over univariable MR: first, it can control for the effects of confounder to improve the accuracy of causal inference. Second, it provides more accurate estimates of causal effects by analyzing pooling effect. Overall, the direction of the causal effect size estimates was consistent between multivariable MR and CAUSE for all analyses undertaken.

## 4 Discussion

This study selected a multitude of sleep traits from the sleep health dimension, circadian characteristic and sleep disturbance, in order to ascertain the correlation and causal relationship between sleep and myopia. Using summary-level data for sleep and myopia from large European populations, our research revealed there was no discernible causal connection between the two variables after accounting for potential confounding factors.

The contention regarding the influence of sleep on myopia has endured for nearly 3 decades, a definitive conclusion remains elusive even now. In 1999, a study initially conducted by Quinn (Quinn et al., 1999) shed light on the relationship between sleep and myopia formation. However, several subsequent studies in Singaporean and British ancestry, have shown no clear relationship between light during sleep and myopia (Gwiazda et al., 2000; Saw et al., 2001; Guggenheim et al., 2003). In recent years, researchers have used more comprehensive and scientific indicators to evaluate sleep, such as sleep duration (Zhou et al., 2015; Ayaki et al., 2016; Jee et al., 2016; Wei et al., 2020; Huang et al., 2022; Lin et al., 2023), chronotype (Liu et al., 2020; Liu et al., 2022; Palumaa et al., 2022; Stafford-Bell et al., 2022) and insomnia (Pan et al., 2019; Mirhajianmoghadam et al., 2021). So, we selected the GWAS data which contained the highest number of SNPs and the largest number of people in these sleep traits, hoping to derive whether genetically predicted sleep is causally related to myopia through a large population-based study.

In the realm of animal experimentation, scientists thought that altering “circadian rhythms (Zhang et al., 2019)” in sleep factors could influence the occurrence and development of myopia (Zhou et al., 2010; Nickla et al., 2017; Patel et al., 2017; Liu et al., 2023). They studied the effects of altering clock genes on eye development using different animal models, specifically observing mice with a deleted retinal-specific clock gene (Bmal1), which resulted in myopia and elongation of the vitreous chamber (Panda et al., 2002; Morton et al., 2005; Stone et al., 2019). Circadian rhythms act as a determinant of chronotype. The term “chronotype” pertains to a bodily inclination reach their peak of cognitive and physical performance towards either morning (commonly referred to as “early lark”) or evening (termed “night owl”), and is also recognized as circadian preference (Root Kustritz et al., 2022). After multiple models exploring genetic associations and causation, we thought differences in chronotype did not lead to differences in the occurrence of myopia (Figure 3B; Supplementary Table S6).

The window for predicting myopia onset is limited (Mutti et al., 2007), which presents a formidable obstacle to precise prediction and prompt intervention in myopia. In an effort to resolve the ongoing controversy between sleep and myopia and to facilitate early myopic clinical prevention, this study used a large sample size from GWASs, which gave it enough statistical validity to estimate causality. In this study the LD Score regression was used to assess the extent to which different genes from the whole genome level are associated with myopia and determined two sleep traits (sleep duration and chronotype). To more objectively assess the causal relationship and to remove bias caused by other sleep phenotypes, multivariable MR analyses was applied and found no evidence for a causal effect of sleep traits on risk of myopia. Finally, the CAUSE which bolstered the credibility of the findings and correct for deviations from false positives supported there was no causal relationship of all sleep traits on myopia data derived from FinnGen and UK Biobank. Combining the above analyses of a range of models, no causal relationship was suggested between sleep and myopia, which is consistent with the results of most observational studies.

However, there are limitations in this study. Lack of cycloplegia in children’s refractive studies can lead to misclassification of myopia by introducing bias towards greater myopia (Flitcroft et al., 2019). The individuals were all of European heritage and the results may appear difficult to extend to different ethnic groups, such as those in East and Southeast Asia, limiting the generalizability of the findings. Also, sleep data were acquired from questionnaires that were self-reported and not objectively tested, which may generate potential bias. Moreover, it has been found that there may be genetic heterogeneity in SNPs associated with sleep duration in adults and children (Marinelli et al., 2016). The present study only demonstrated that sleep duration was not causally associated with myopia in adults of European ancestry. Further studies on children’s sleep duration-associated SNPs need to be conducted with their age-level-matched GWAS databases for myopia. At last, in database adoption, there is some unavoidable population sample overlap to the extent that we allow it (Burgess et al., 2016). (We estimated in https://sb452.shinyapps.io/overlap/, and the results indicated that the sample overlap was so small that statistical efficacy was not affected (Pierce and Burgess, 2013), Supplementary Table S8).

Overall, this study had neither evidence to support a protective or deleterious effect of genetically predicted sleep traits on myopia. further largescale or longitudinal studies are required to investigate the causal relationship between sleep and myopia. In addition, the latest data from large-scale genetic studies and “fine grained” approaches of monitoring sleep (Oza, 2023; Türker et al., 2023) can also be used for further studies. This study suggests that future research could design long follow-up studies to track changes in lifestyle habits and changes in visual acuity in different age groups, providing dynamic data on the development of myopia and helping to better understand its etiology and prevention methods. Although the findings suggest that sleep characteristics may not be the primary determinant of myopia development, it is still important to maintain a good and healthy sleep schedule with children. Further research is needed to explore other potential factors contributing to the development of myopia.

## 5 Conclusion

Our research revealed there was no discernible causal connection between sleep and myopia in European ancestry after accounting for potential confounding factors.

## Data Availability

The datasets presented in this study can be found in online repositories. The names of the repository/repositories and accession number(s) can be found in the article/Supplementary Material.
