# Supplementary material for: Genetic correlation and causal relationship between sleep and myopia: a mendelian randomization study
Source: Front Genet. 2024 Jul 9;15:1378802. doi: 10.3389/fgene.2024.1378802 (PMC11263174; doi:10.3389/fgene.2024.1378802)

Contents

A Results of CAUSE for sleep duration and myopia(UK Biobank).

B Results of CAUSE for chronotype and myopia(UK Biobank).

C Results of CAUSE for insomnia and myopia(UK Biobank).

D Results of CAUSE for sleep duration and myopia(FinnGen).

E Results of CAUSE for chronotype and myopia(FinnGen).

F Results of CAUSE for insomnia and myopia(FinnGen).

A

	model1	model2	delta_elpd	se_delta_elpd	z	p
1	null	sharing	0.360	0.23	1.50	0.94
2	null	causal	0.270	1.30	0.21	0.58
3	sharing	causal	-0.087	1.10	-0.08	0.47

model	gamma	eta	q
Sharing	NA	0.03 (-0.13, 0.24)	0.04 (0, 0.25)
Causal	0.01 (0, 0.02)	0.01 (-0.14, 0.19)	0.04 (0, 0.25)

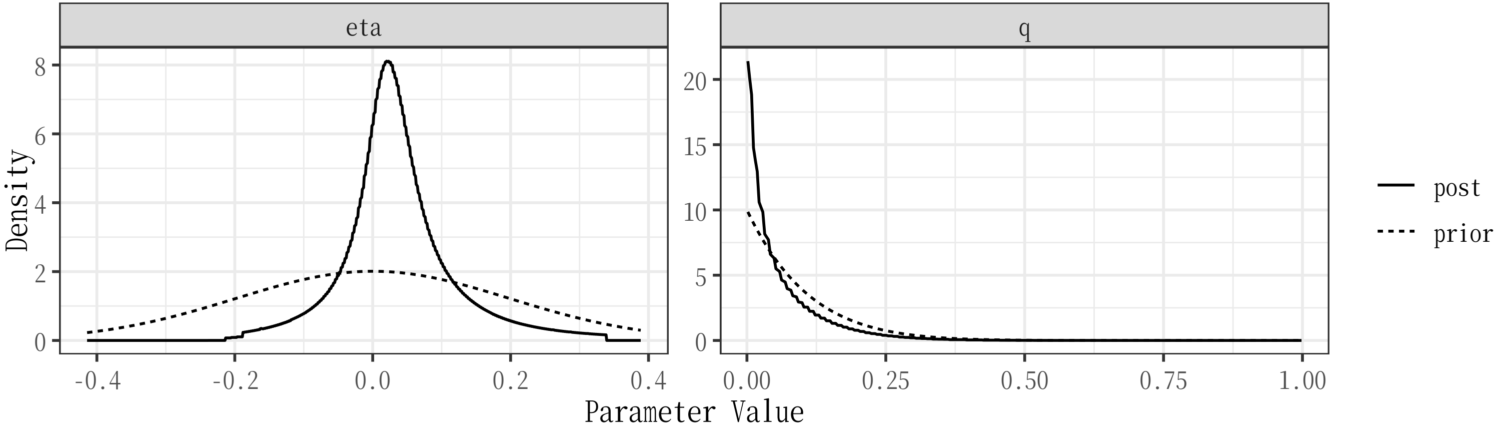

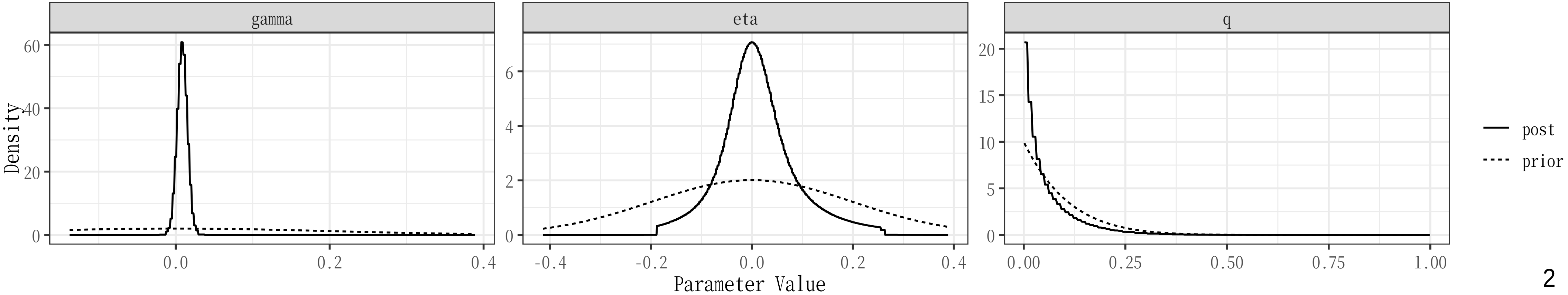

B

	model1	model2	delta_elpd	se_delta_elpd	z	p
1	null	sharing	0.40	0.11	3.7	1.00
2	null	causal	0.91	0.72	1.3	0.90
3	sharing	causal	0.51	0.63	0.8	0.79

model	gamma	eta	q
Sharing	NA	-0.01 (-0.09, 0.1)	0.04 (0, 0.24)
Causal	0 (-0.01, 0.01)	0 (-0.08, 0.11)	0.04 (0, 0.25)

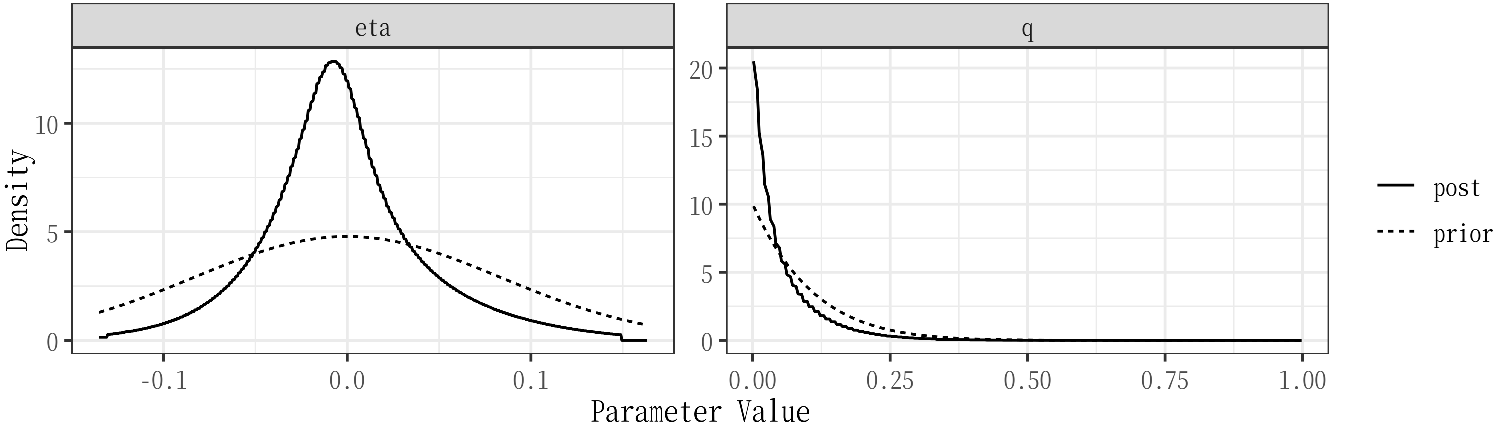

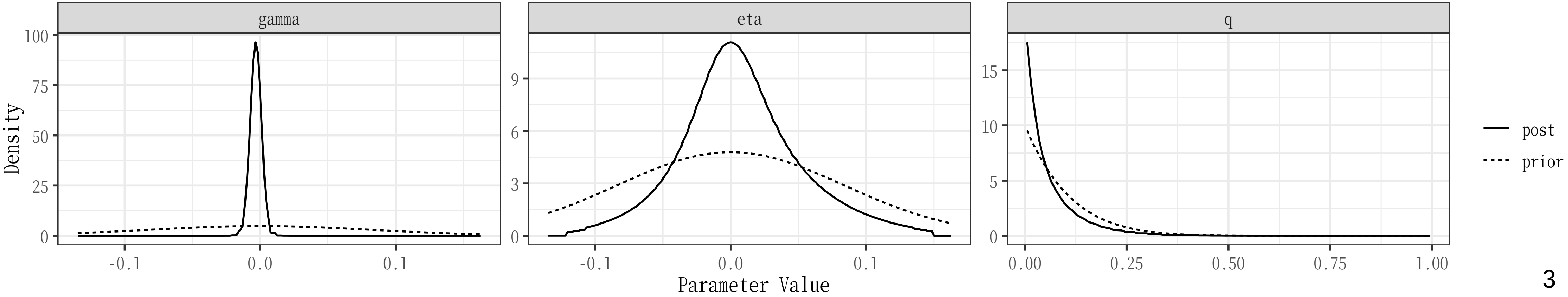

C

	model1	model2	delta_elpd	se_delta_elpd	z	p
1	null	sharing	0.060	0.05	1.200	0.88
2	null	causal	-0.058	0.59	-0.098	0.46
3	sharing	causal	-0.120	0.55	-0.210	0.42

model	gamma	eta	q
Sharing	NA	0 (-0.02, 0.02)	0.06 (0, 0.3)
Causal	-0.01 (-0.02, 0)	0 (-0.02, 0.02)	0.06 (0, 0.3)

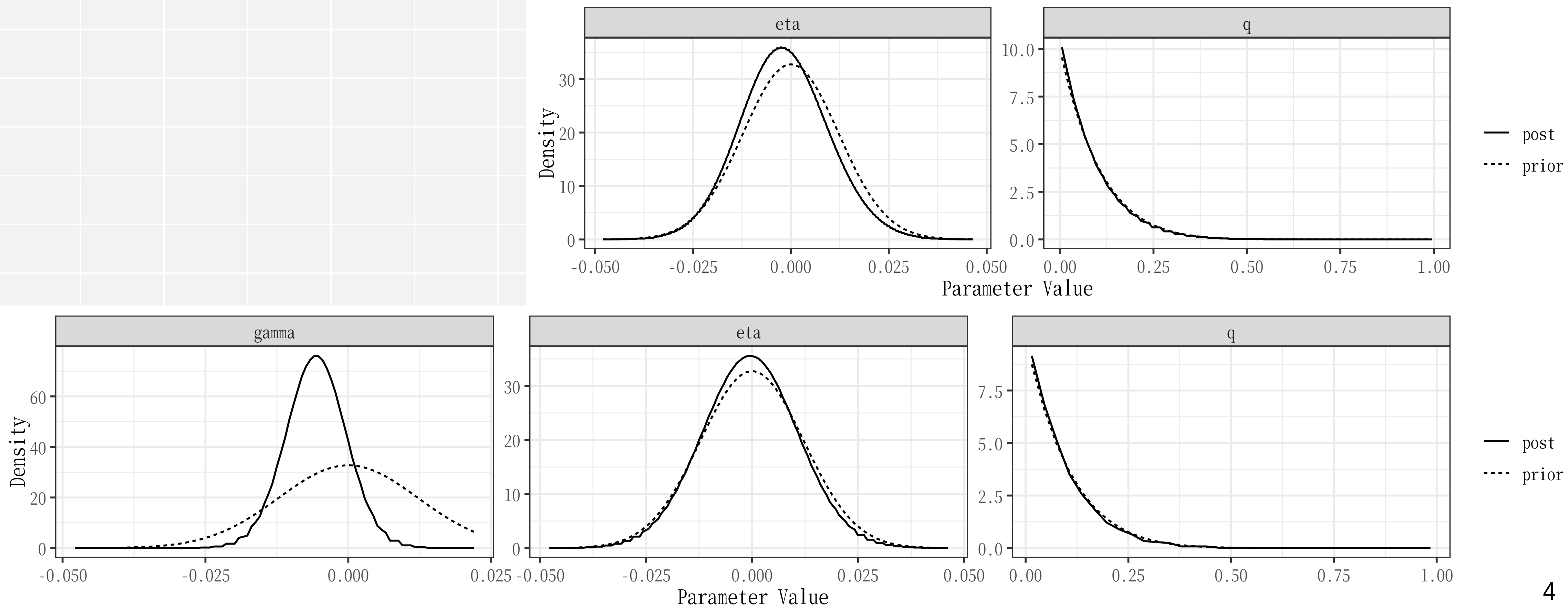

D

	model1	model2	delta_elpd	se_delta_elpd	z	p
1	null	sharing	0.11	0.43	0.25	0.60
2	null	causal	-0.38	1.70	-0.23	0.41
3	sharing	causal	-0.49	1.30	-0.39	0.35

model	gamma	eta	q
Sharing	NA	-1.43 (-6.93, 5.48)	0.05 (0, 0.28)
Causal	-0.43 (-0.97, 0.12)	0.07 (-6.25, 7.01)	0.04 (0, 0.26)

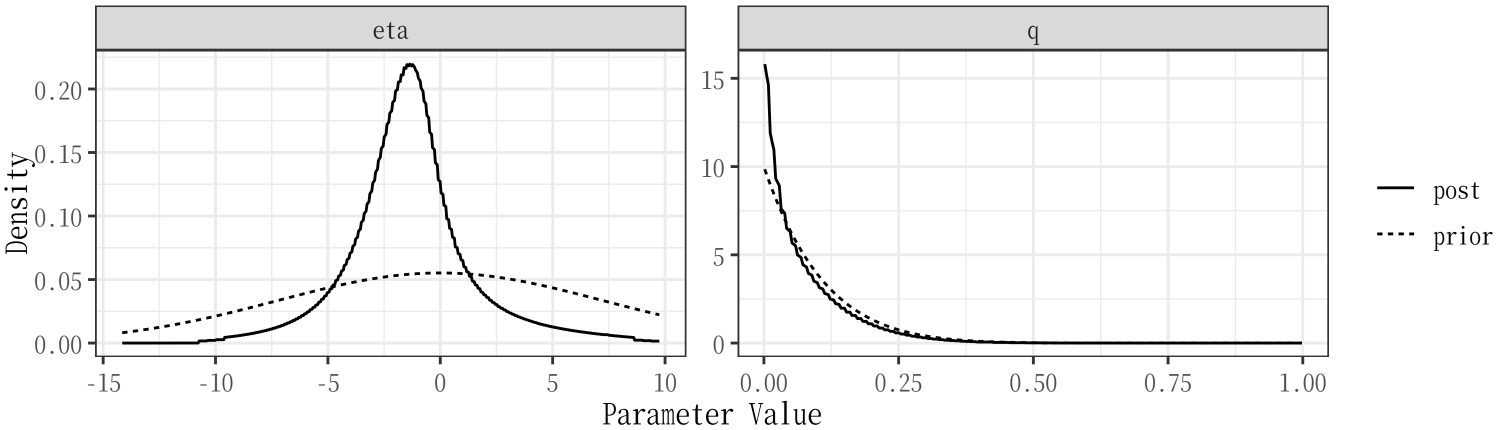

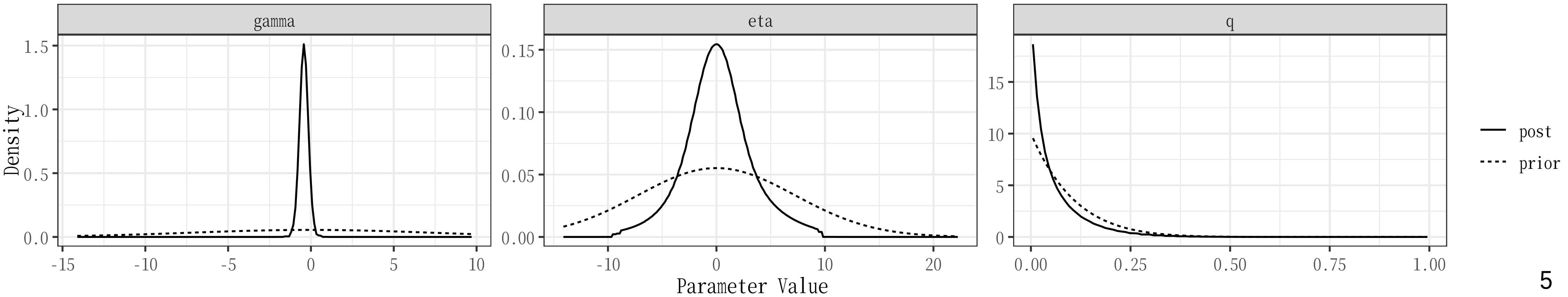

E

	model1	model2	delta_elpd	se_delta_elpd	z	p
1	null	sharing	0.41	0.37	1.1	0.87
2	null	causal	0.85	1.10	0.8	0.79
3	sharing	causal	0.44	0.73	0.6	0.73

model	gamma	eta	q
Sharing	NA	-0.82 (-6.04, 5.78)	0.03 (0, 0.23)
Causal	-0.13 (-0.5, 0.22)	0.08 (-5.5, 6.42)	0.04 (0, 0.26)

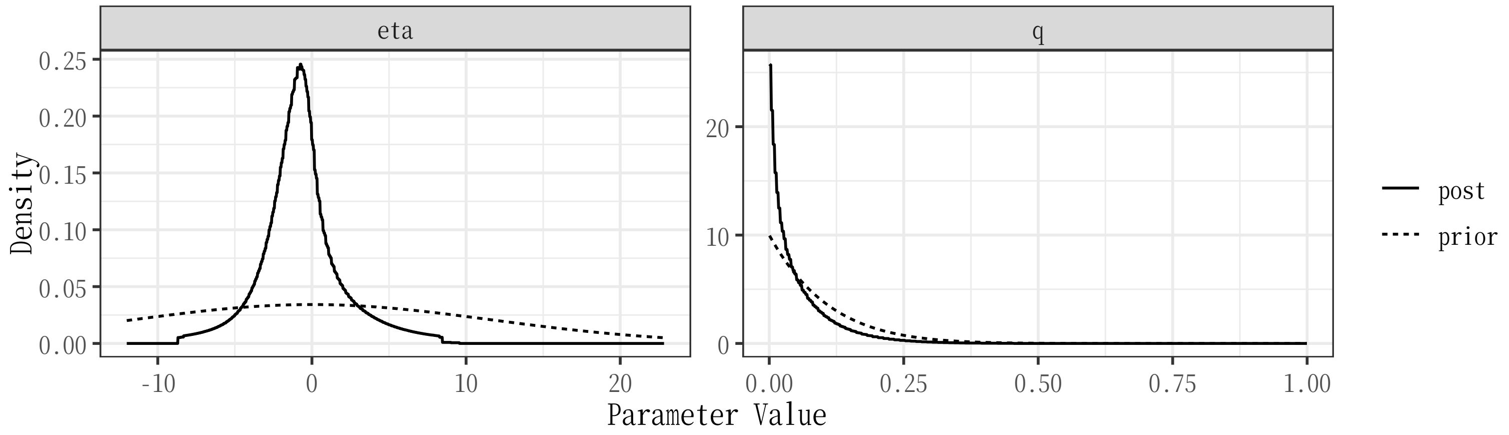

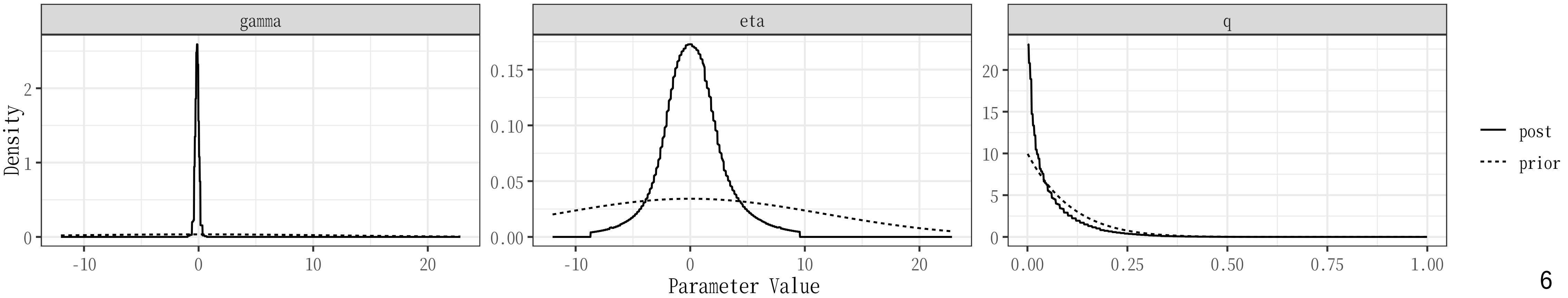

F

	model1	model2	delta_elpd	se_delta_elpd	z	p
1	null	sharing	0.26	0.13	2.1	0.98
2	null	causal	1.00	0.42	2.4	0.99
3	sharing	causal	0.75	0.32	2.3	0.99

model	gamma	eta	q
Sharing	NA	-0.02 (-1.51, 1.52)	0.05 (0, 0.25)
Causal	-0.02 (-0.23, 0.17)	0 (-1.5, 1.51)	0.05 (0, 0.26)

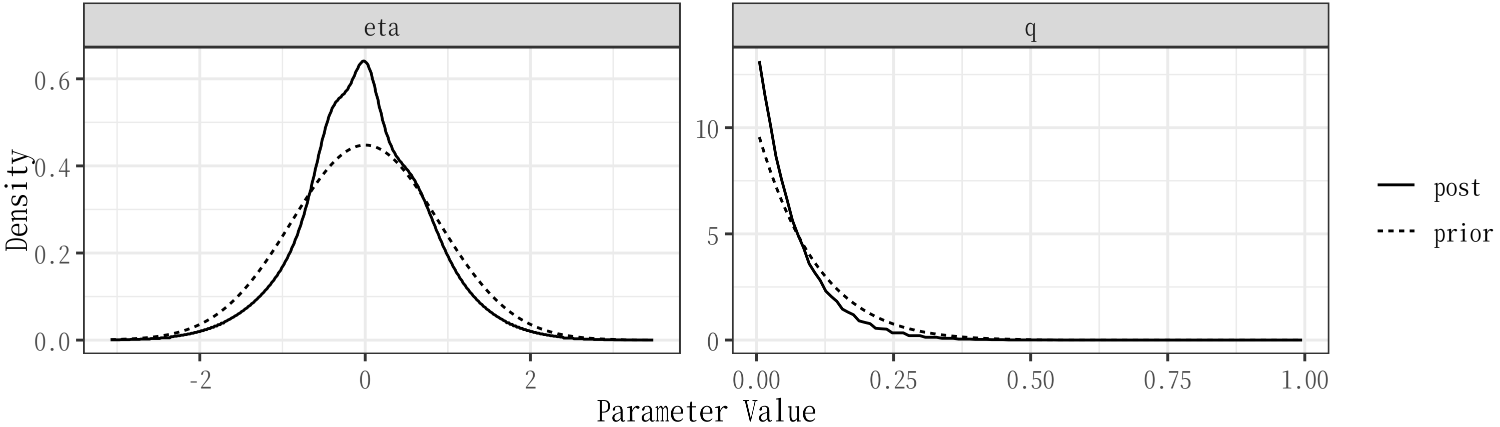

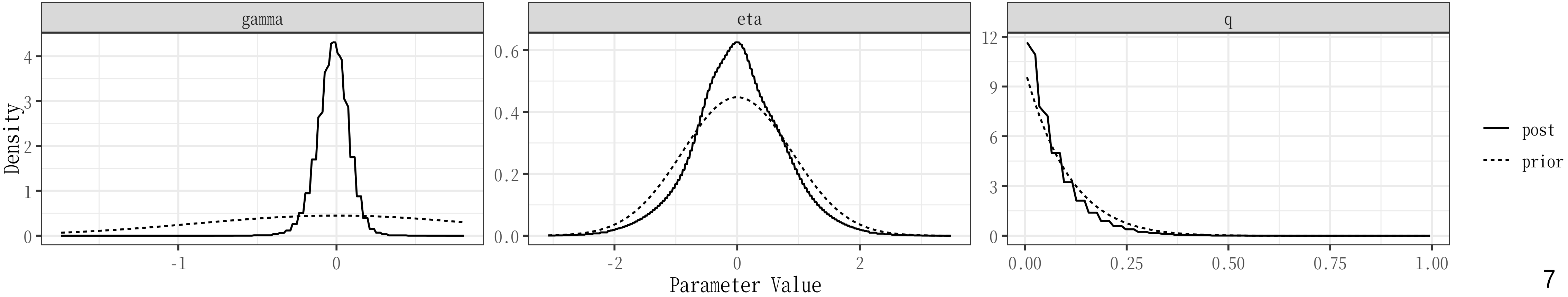

Supplement: Supplementary file 5 [file DataSheet1.PDF]
